# Similar Intracellular Location and Stimulus Reactivity, but Differential Mobility of Tailless (*Vicia faba)* and Tailed Forisomes (*Phaseolus vulgaris*) in Intact Sieve Tubes

**DOI:** 10.1371/journal.pone.0143920

**Published:** 2015-12-01

**Authors:** Alexandra C. U. Furch, Stefanie V. Buxa, Aart J. E. van Bel

**Affiliations:** 1 Institute of General Botany and Plant Physiology, Friedrich-Schiller-University, Jena, Germany; 2 Research Centre for BioSystems, Land Use and Nutrition, Institute of Phytopathology and Applied Zoology, Justus-Liebig University, Giessen, Germany; 3 Plant Cell Biology Research Group, Institute of General Botany, Justus-Liebig University, Giessen, Germany; Beijing Forestry University, CHINA

## Abstract

Sieve elements of legumes contain forisomes—fusiform protein bodies that are responsible for sieve-tube occlusion in response to damage or wound signals. Earlier work described the existence of tailless and tailed forisomes. This study intended to quantify and compare location and position of tailless (in *Vicia faba*) and tailed (in *Phaseolus vulgaris)* forisomes inside sieve elements and to assess their reactivity and potential mobility in response to a remote stimulus. Location (distribution within sieve elements) and position (forisome tip contacts) of more than altogether 2000 forisomes were screened in 500 intact plants by laser scanning confocal microscopy in the transmission mode. Furthermore, we studied the dispersion of forisomes at different locations in different positions and their positional behaviour in response to distant heat shocks. Forisome distribution turned out to be species-specific, whereas forisome positions at various locations were largely similar in bushbean (*Phaseolus*) and broadbean (*Vicia*). In general, the tailless forisomes had higher dispersion rates in response to heat shocks than the tailed forisomes and forisomes at the downstream (basal) end dispersed more frequently than those at the upstream end (apical). In contrast to the tailless forisomes that only oscillate in response to heat shocks, downstream-located tailed forisomes can cover considerable distances within sieve elements. This displacement was prevented by gentle rubbing of the leaf (priming) before the heat shock. Movement of these forisomes was also prohibited by Latrunculin A, an inhibitor of actin polymerization. The apparently active mobility of tailed forisomes gives credence to the idea that at least the latter forisomes are not free-floating, but connected to other sieve-element structures.

## Introduction

Sieve elements of legumes contain forisomes [1], fusiform giant macromolecular conglomerates up to a 100 microns in length [2] without—tailless—or with “tails” at the tips—tailed—[2–4]. Forisomes seem to be composed of spindle-like subunits [5] designated as forisomettes [6]. Their substructure exhibits a matrix of regularly ordered building blocks e.g. [1, 5, 7] which are largely of a proteinaceous nature. These proteins belong to the SEO family [8] which is presumably involved in sieve-element occlusion (SEO) and seems widespread among dicotyledons [9–13].

The capacity to sieve-element occlusion is reflected by a striking property of forisomes: they are able to disperse and re-condense in response to changes in the sieve-element internal milieu [1]. Studies using isolated forisomes obtained via sophisticated isolation methods [14] visualized the dimensions of these conformation changes [15, 16]. Ca^2+^ induced a 3 to 6-fold volume increase within 10 to 15 seconds [14, 16] (S4 Fig). Isolation of forisomes further enabled *in vitro* testing of their biotechnological potential for microfluidics [16] and other applications [17]. In this way, the occlusion ability of dispersed forisomes has been demonstrated using silicon-based tubular systems with sieve-element diameters [16].

Both *in situ* [1] as well as *in vitro* [14, 16] forisomes disperse in response to Ca^2+^ supply. After withdrawal of Ca^2+^-ions, forisomes resume the original, condensed conformation [1, 14, 16, 18]. The tails do not react to Ca^2+^ supply and, therefore, may be of a different composition or architecture [8, 13, 19]. The reversible conformation changes reflect forisome behaviour in response to sieve-element injury [20, 21]. In case of damage, apoplasmic Ca^2+^-ions are flooding the sieve-element lumen, which results in sieve-plate occlusion by forisome dispersion probably in combination with protein agglutination [20]. Abrupt and massive Ca^2+^ influx into lumina of intact sieve elements can also be achieved by vigorous stimuli [20–22]. Local cold shocks [22] and remote heat shocks [20, 21] associated with abrupt Ca^2+^ influx bring about forisome dispersion.

Forisome dispersion is taken as an indication that Ca^2+^ has surpassed an activation threshold inside sieve elements after wounding or during passage of electropotential waves [21, 23, 24]. It appears that forisome only disperse if the Ca^2+^-concentration inside the sieve element rises above 50 μmol [21]. In intact sieve elements, such high Ca^2+^-concentrations may only be reached at certain hotspots. Calcium hotspots may be created by a concerted gating of voltage-dependent and mechano-sensitive Ca^2+^-permeable channels in the sieve-element plasma membrane and Ca^2+^-dependent Ca^2+^-channels at the membranes of the ER-stacks [23, 25]. This proposal is in agreement with the observation that forisomes are most reactive to distant stimuli in sieve-element areas where Ca^2+^ channels are densely aggregated e.g. in the vicinity of the sieve plates.

There is some evidence that the forisome tips are in contact with or even attached to sieve-element membrane structures [21], which opens the interesting perspective that they may be kept in place near Ca^2+^-hotspots for optimal functioning This idea concurs with the observation that the forisome position is an important determinant of reactivity. Forisomes with both tips in contact with the plasma membrane region dispersed to distant heat shocks to a greater extent than those with one tip or neither of them against the plasma membrane [21]. Supportive to potential forisome anchoring is that several forisomes are located at the sieve plate at the upstream, apical end of the sieve element [15] and not at the downstream, basal end as to be expected for free-floating forisomes dragged along with mass flow.

In the first place, therefore, the reactivity of upstream *Vicia* forisomes in different positions to distant stimuli needed further verification by situ examination in intact plants. Furthermore, location, position, and reactivity to distant heat shocks (expressed as dispersion) of the tailless forisomes in *Vicia faba* (broadbean) and the tailed forisomes in *Phaseolus vulgaris* (bushbean) were compared using water immersion objectives in confocal laser scanning microscopy in the transmission mode. Finally, we studied positional changes and eventual mobility of both forisome types in response to distant heat shocks.

## Material and Methods

### Plant Material

Plants of *Vicia faba–*broadbean—cv Witkiem major (Nunhems Zaden BV, Haelen, The Netherlands) and *Phaseolus vulgaris–*bushbean—cv Hildora (Hild, Marbach, Germany) were cultivated in pots in a greenhouse at temperature varying between 20–30°C, 60–70% relative humidity, and a 14/10 h light/dark regime. Supplementary lighting (model SONT Agro 400 W; Philips Eindhoven, the Netherlands) was used to give an irradiance level of 200–250 μmol^-2^ sec^-1^ at the plant apex. Plants were used 4–7 weeks after germination, in the vegetative phase just before flowering. The plants were transferred in protective boxes from the greenhouse to the laboratory the day before the experiments and stayed there overnight at room temperature of about 20°C to standardize the conditions.

### Preparation of intact plants for observation by confocal laser scanning microscopy

For *in vivo* observation of sieve tubes, cortical cell layers were removed to create an observation window on the phloem at the abaxial side of the main vein of a mature leaf still attached to the intact plant. The cortical tissue was locally removed by manual paradermal slicing with a fresh razor blade, while avoiding damage to the phloem [26]. The bare-lying tissue was immediately submersed in bathing medium containing 2 mol m^-3^ KCl, 1 mol m^-3^ CaCl_2_, 1 mol m^-3^ MgCl_2_, 50 mol m^-3^ mannitol and 2.5 mol m^-3^ MES/NaOH buffer, pH 5.7. The leaf was fixed onto a microscope slide with two strips of double-sided adhesive tape and mounted on the stage of a confocal laser scanning microscope. After manipulation of the plants, a resting period of at least one hour was taken to restore the ground situation in order to avoid visual misinterpretations. Intactness of sieve elements was checked microscopically via a water immersion objective in the dipping mode by verifying the presence of condensed forisomes. After verification of phloem viability, the leaf tip was burnt carefully by a focused lighter flame, which caused a heat shock. The distance between leaf tip and observation window was 3–4 cm. So-called “priming” of *Phaseolus vulgaris* plants was elicited by delicate rubbing of the leaf tip to be burnt between two finger tips without causing damage.

The actin-polymerization inhibitor latrunculin A (LatA) was prepared as a stock solution dissolved in DMSO and diluted in bathing medium to give a final working concentrations of 500 nM LatA in 0.5mM (0.01% [v/v]) DMSO. LatA was purchased from Invitrogen (Carlsbad, California, USA).

### Confocal laser scanning microscopy

Forisomes and phloem tissue were imaged by confocal laser scanning microscopy using a Leica TCS 4D and SP2 (Leica Microsystems, Heidelberg, Germany) in the black-and-white mode. The phloem tissues were observed using a water immersion objective (HCX APO L40x0.80 W U-V-l objective, Leica, Heidelberg, Germany) in the dipping mode. Digital images were processed with Adobe® PhotoShop to optimize brightness and contrast.

### Statistical analyses

Statistical analyses were carried out using SPSS® (IBM® SPSS® Statistics 22). Statistical significance of the differences in forisome location and position were examined by Chi-Square tests with Bonferroni correction. Significance level is defined as the two-sided asympototic significance of chi-square statistics (p<0.05). Forisome reactivity was examined by one-way analysis of variance (ANOVA) with a Bonferroni correction to increase the rigorosity of significance [27] and given as the Welch’s F ratio (p<0.05). The correlation between forisome dispersion and re-condensation times was evaluated by use of Pearson product-moment correlation coefficient, which is considered significant if p<0.05.

## Results

### Location and position of tailless and tailed forisomes in sieve elements

**Fig 1:**
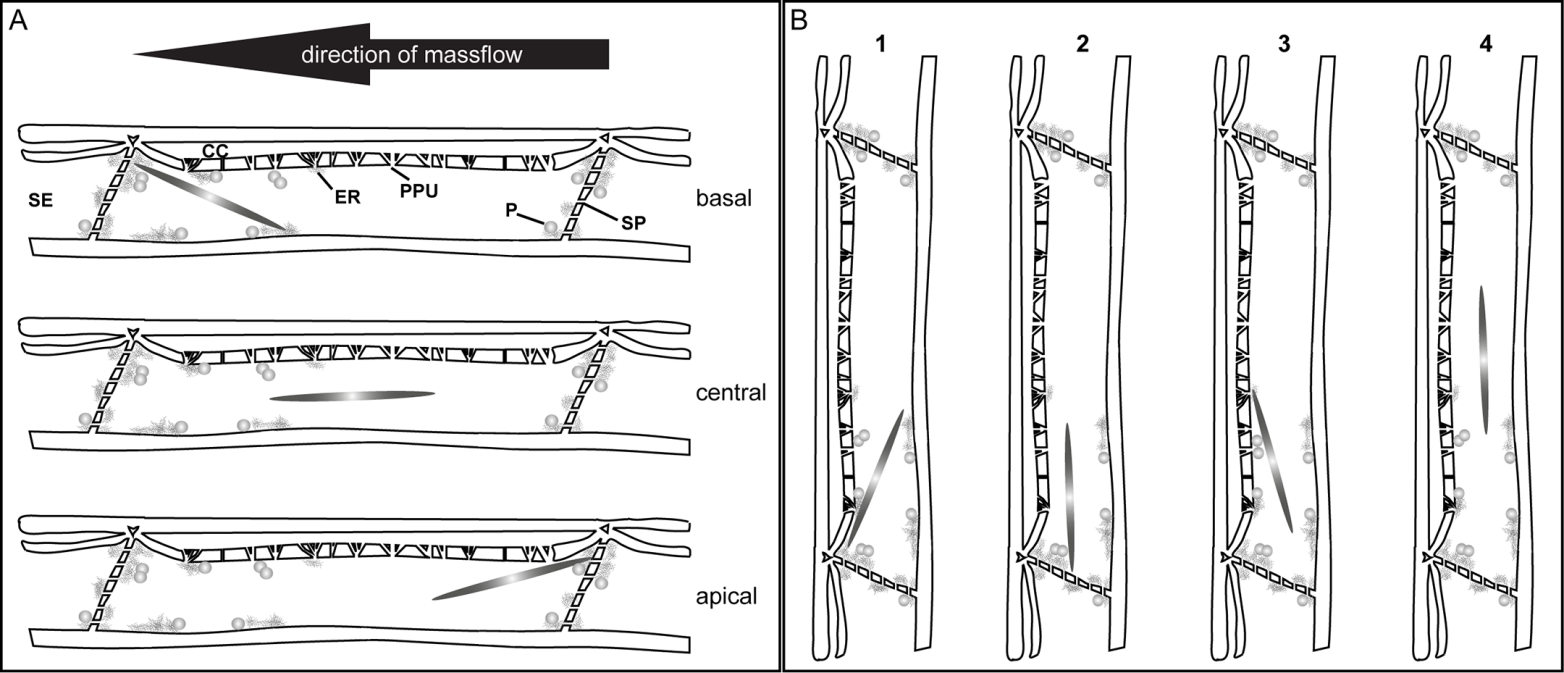
Forisome locations and positions inside sieve elements. A. Basal (downstream), central and apical (upstream) locations. B. Position1, Forisome tips in contact with the plasma membrane lining the sieve plate and the parietal plasma membrane, position 2 one tip in contact with the plasma membrane lining the sieve plate, the other is free-floating in the sieve-element lumen, position 3 one tip in contact with the parietal plasma membrane, position, the other is free-floating in the sieve-element lumen, position 4 no apparent tip contacts.

In this study, location and position of more than 2000 forisomes in at least 500 intact plants were screened. According to the nomenclature adopted here, forisomes are located in basal, central and apical parts of sieve elements (Fig 1A) in the 4 following positions (Fig 1B): one forisome tip is in contact with the plasma membrane section lining the sieve plate, the other with the parietal plasma membrane of the sieve element (position 1), one forisome tip is in contact with the plasma membrane section lining the sieve plate, the other tip is free-floating (position 2), one tip is in contact with the parietal plasma membrane, the other is free-floating (position 3), or no apparent contacts between forisome tips and plasma membrane (position 4).

**Fig 2:**
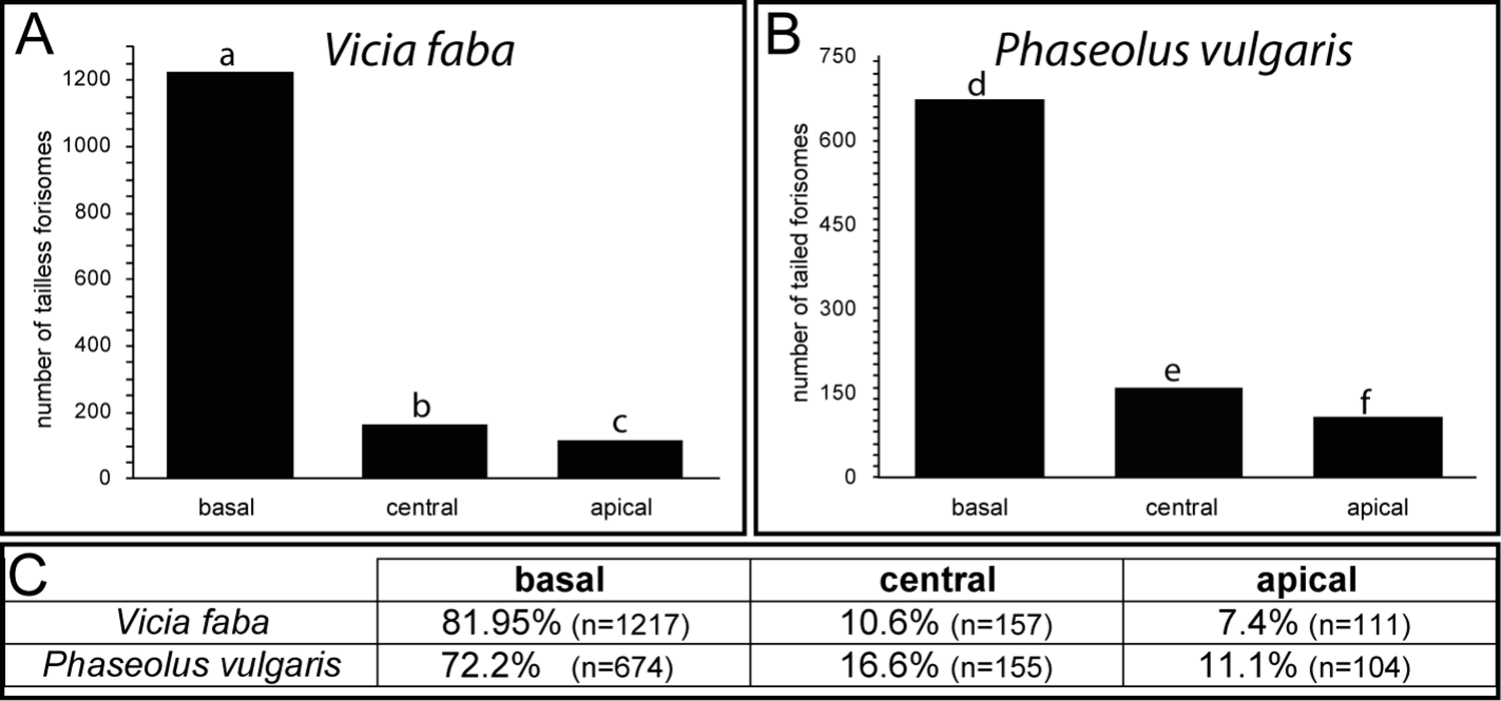
Absolute numbers and percentage of forisome locations (basal, central, apical) in sieve elements of A. *Vicia faba* and B. *Phaseolus vulgaris*. Different letters indicate significant differences (p < 0.05) between locations and plant species.

The location of tailless (*Vicia faba*) and tailed (*Phaseolus vulgaris*) forisomes inside the sieve elements was seemingly similar at first sight (Fig 2A and 2B). After statistical treatment, however, the distribution of forisomes was significantly different between the two species (Fig 2A and 2B). In *Vicia faba*, 18% of the forisomes was not located at the basal, downstream end of the sieve element (Fig 2A), in *Phaseolus vulgaris* 27.7% (Fig 2B). Within the species forisome distribution was non-random (p<0.05) with the downstream location strongly favoured both in broadbean and in bushbean (Fig 2A and 2B) which confirms an earlier report that forisomes are mostly located at the basal end of *Vicia* sieve elements [15].

**Fig 3:**
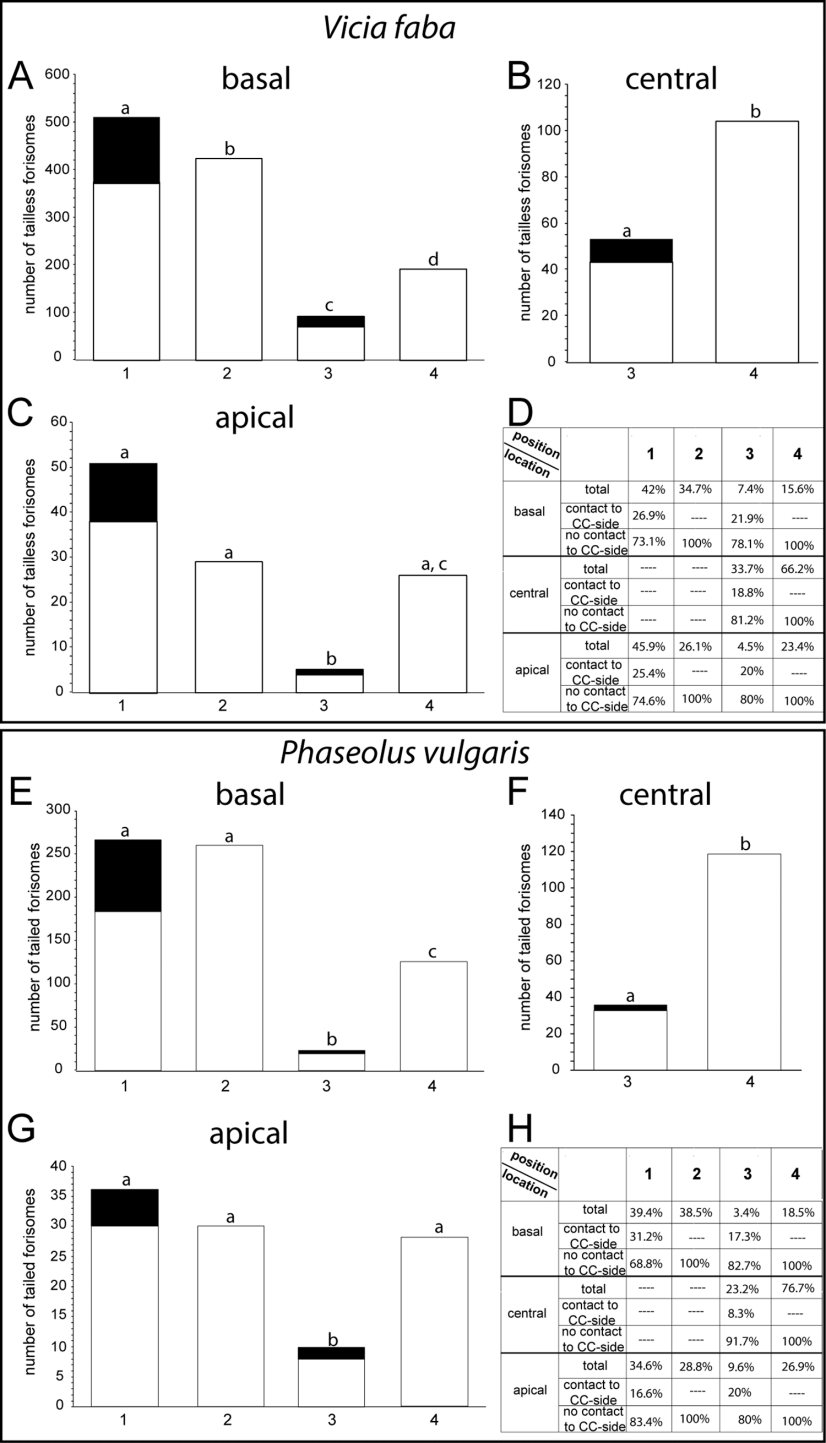
Forisome positions at diverse sieve-element locations in *Vicia faba* (A-D) and *Phaseolus vulgaris* (E-H). A. and E. basal (downstream); B. and F. central; C. and G. apical (upstream). D. and H. Percentage of forisomes at each location and position. The black areas indicate forisomes in contact with the sieve-element side facing the companion cell. Different letters indicate significant differences between the positions of forisomes.

Positions of forisomes at various locations were determined using Z-stacks of transmission pictures (S1 Fig and Fig 2). The respective positions of forisomes at diverse locations were almost identical in *Vicia* (Fig 3A–3D) and *Phaseolus* (Fig 3E–3H). Statistical analysis showed no significant differences of forisome position between *Vicia* and *Phaseolus* (p<0.05). Most of the forisomes, both at the downstream and upstream side in *Vicia* (Fig 3A–3C) and *Phaseolus* (Fig 3E–3G), were in position 1 followed by the positions 2, 4, and 3, respectively. About 30% of the forisomes, both at the downstream and upstream ends, were in contact with the sieve-element membrane facing the companion cell (Fig 3D and 3H), significantly favouring contact to plasma membrane facing parenchyma cells (p < 0.05).

### Dispersion of tailless and tailed forisomes in response to distant heat shocks in intact plants

**Fig 4:**
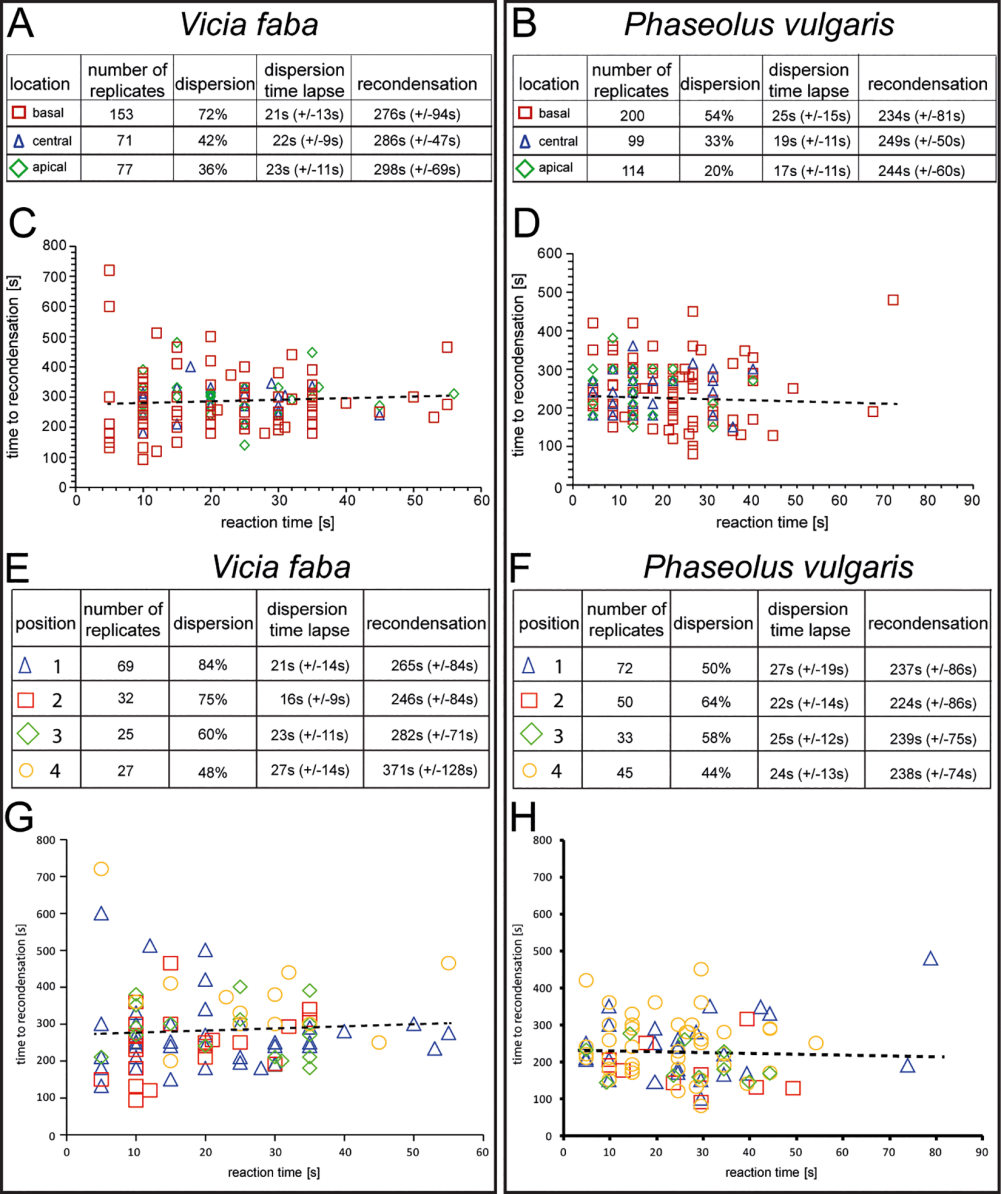
Forisome reactivity (dispersion and re-condensation) in response to a remote heat stimulus at different locations in A. and C. *Vicia faba* and B. and D. primed *Phaseolus vulgaris* and different positions at the basal (downstream) location (E. and G. *Vicia faba* and F. and H. *Phaseolus vulgaris*. The stippled line indicates the relationship between dispersion and re-condensation time.

After application of a distant heat shock, we examined forisome reactivity (expressed as dispersion) at the respective locations and positions (Fig 4). For reasons of brevity, we have omitted images of forisome dispersion and recondensation, which have been amply documented in the literature. In vitro reversibility of forisomes of the present lot was also tested and the volumes of the dispersed foriomes were in the order of the values reported. While the forisomes in *Vicia* readily dispersed in response to a heat shock after recovery from tissue preparation, forisome dispersion in *Phaseolus* often required a slight touch of the leaf tip cf. [28] prior to the burning stimulus. Without this mechanical stimulus only 26% of the *Phaseolus* forisomes dispersed. After the manual “priming touch”, forisomes dispersed dependent on location (20 to 54%; Fig 4B) or position at the downstream end (44 to 64%; Fig 4F). Basal and apical *Phaseolus* forisomes were significantly (p < 0.05) less dispersive than *Vicia* forisomes at identical locations (Fig 4A and 4B).

The forisomes at the downstream end showed significantly higher reactivity rates than those at other locations (p<0.05) both in *Vicia* (Fig 4A) and in *Phaseolus* (Fig 4B). The average reaction times (time lapse between stimulus and dispersion) were the lowest for basal forisomes in *Vicia* (Fig 4A and 4B) and apical forisomes in *Phaseolus* (Fig 4C and 4D), albeit not to a significant extent (p>0.05). A significant correlation neither exists between the dispersion and re-condensation times both in *Vicia* and in *Phaseolus* forisomes at all locations (Fig 4C and 4D; correlation coefficient R < 0,005).

Taking the positions of solely the basal forisomes into account (Fig 4E and 4F), *Vicia* forisomes in the positions 1 and 2 showed significantly higher reactivity rates to heat shocks (p<0.05), followed by those in position 3 and 4, in this order (Fig 4E). This contrasted the situation in *Phaseolus*, where the forisomes in position 2 were significantly most reactive, followed by those in position 3, 1 and 4 (Fig 4F). A significant effect of the forisome position on the reaction times was not found in both plants (p<0.05). Again, a statistically significant correlation between reaction times and re-condensation times could not be shown (Fig 4G and 4H; correlation coefficient R< 0,005). In conclusion, locations, positions and reactivity patterns of tailed and tailless forisomes were largely similar in *Vicia* and “primed” *Phaseolus plants*,

### Differential mobility of tailless and tailed forisomes

**Fig 5:**
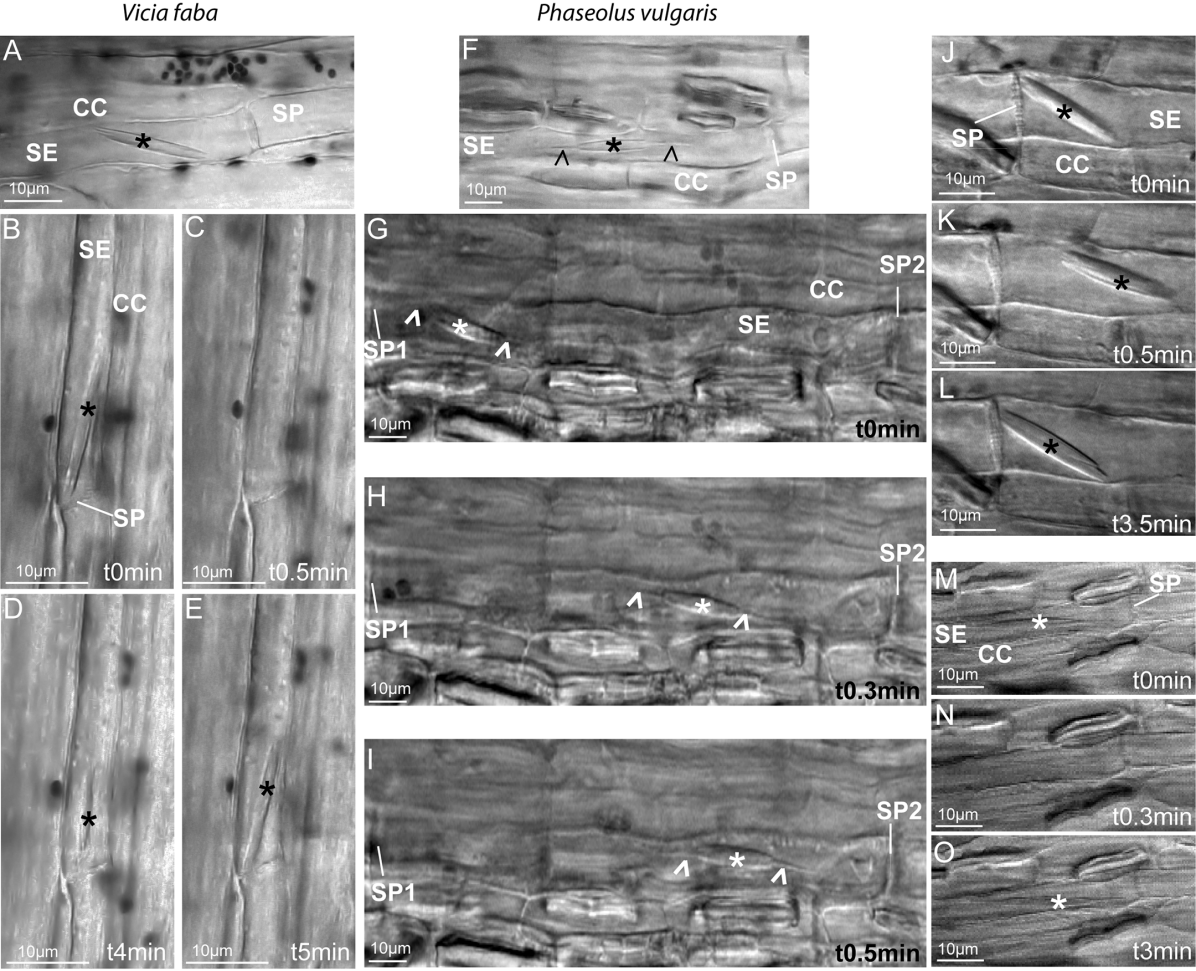
Reactions and change of forisome position in *Vicia faba* (A-E) and forisome location in *Phaseolus vulgaris* sieve elements (F-O) in response to remote heating (forisomes are marked by asterisks). A. *Vicia faba* forisome (upstream position 1). B-E *Vicia faba* forisome (downstream position, position1). B. Initial position at an angle of about 10° to the longitudinal axis, C. Dispersion in response to a remote heat shock, D. Re-condensation, 4 min after the stimulus, position parallel to the longitudinal axis, E. 5 min after stimulus, position at an angle of about 10° to the longitudinal axis. F. *Phaseolus vulgaris* forisome (upstream position). G-I Long-distance ovement of a non-primed condensed Phaseolus vulgaris forisome from the downstream position 1 to the upstream position 4, J-L Short-distance movement of a non-primed condensed *Phaseolus vulgaris* forisome from the downstream position 1 (J.), via the central position 4 (K) back to the original downstream position 1 (L). M-O No position change of a dispersed *Phaseolus vulgaris* forisome. Direction of flow in G to O from right to left.

As for the forisome mobility, we observed a spectacular difference between *Vicia* and primed *Phaseolus* plants on the one hand and non-primed *Phaseolus* plants on the other. In the latter group, forisomes moved through the sieve elements. Another set of plants was screened for further studies on forisome mobility (Fig 5). After dispersion, *Vicia* forisomes stayed in place and took their initial position after re-condensation despite slight local oscillations (Fig 5B–5E). The same held for dispersed forisomes in “primed” *Phaseolus plants* (Fig 5M–5O). However, when *Phaseolus* plants were exposed to a heat shock without prior mechanical “priming”, 40% of the basal forisomes (37 from 95, Table 1) did not disperse, but travelled longer distances to find a new position (Fig 5G–5I) or moved shorter distances before they returned to their initial position (Fig 5J–5L). Apical forisomes never moved (n = 48, Table 1); movement of central forisomes was rarely observed (2 from 34, Table 1).

**Table 1:** Forisome reactivity in response to remote burning of intact non-primed *Phaseolus vulgaris* plants as related to its location. “Reaction” includes both dispersion or movement.

	number of replicates	dispersion [%]	movement [%]	no reaction [%]	reaction [%]
**basal**	**95**	**32** (n = 30)	**39** (n = 37)	**29** (n = 28)	**69** (n = 67)
**central**	**34**	**26** (n = 9)	**6** (n = 2)	**68** (n = 23)	**32** (n = 11)
**apical**	**48**	**19** (n = 9)	**0**	**81** (n = 39)	**19** (n = 9)
**total**	**177**	**26** (n = 48)	**15** (n = 39)	**59** (n = 90)	**40** (n = 87)

**Fig 6:**
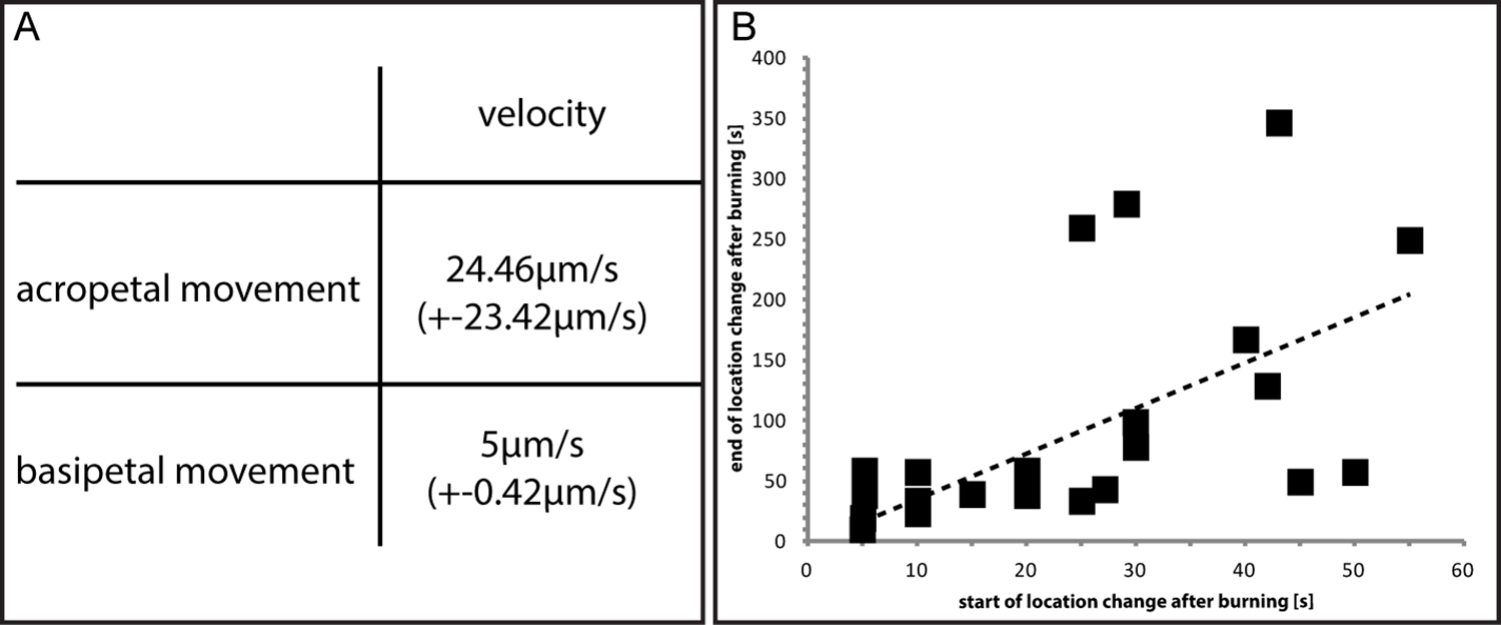
A. Average acropetal and subsequent basipetal movement velocities of originally downstream located condensed *Phaseolus vulgaris* forisomes after a distant heat stimulus in non-primed plants. B. Response lag times of location changes of forisomes in *Phaseolus* vulgaris and the distances of movement. The stippled line indicates the relationship between dispersion and re-condensation time.

Breaking down the data for the basally located forisomes in *Phaseolus* (n = 95, Table 1) according to their position demonstrates that almost exclusively those in position 1 reacted to burning by movement (Table 2). The forisomes in other positions (2–4) reacted by dispersion or did not react at all (Table 2). In case of displacement, basal *Phaseolus* forisomes moved in acropetal direction at an average speed of 24.5 μm/s and returned in basipetal direction at an average speed of 5 μm/s (Fig 6A). As only the slower movements were more suitable for photographic registration, the pictures here present forisomes in slower movement range with a speed of about 2 μm/s (Fig 5F–5I). The reaction times widely varied between the forisomes (Fig 6B). There is a positive correlation between the onset of the movement and the distance covered within the sieve element i.e. longer reaction times are correlated with farther movements (Fig 6B; correlation coefficient R = 0.677, p = 0.01).

**Table 2:** Reactivity of basal forisomes in response to remote burning of intact *Phaseolus vulgaris* plants as related to its position. “Reaction” includes both dispersion and movement.

	number of replicates	dispersion [%]	movement [%]	no reaction [%]	reaction [%]
**1**	**45**	**24** (n = 11)	**71** (n = 32)	**4** (n = 2)	**96** (n = 43)
**2**	**19**	**42** (n = 8)	**11** (n = 2)	**47** (n = 9)	**53** (n = 10)
**3**	**21**	**43** (n = 9)	**5** (n = 1)	**52** (n = 11)	**48** (n = 10)
**4**	**10**	**20** (n = 2)	**20** (n = 2)	**60** (n = 6)	**40** (n = 4)

### Effects of Latrunculin A on forisome conformation and movement

Mobility of forisomes may be associated with the actin network identified in SEs [29]. To investigate the involvement of actin in forisome displacement, its dispersion and movement were investigated after application of 500 nM Latrunculin A (LatA), an inhibitor of actin filament polymerization (see [29] for interference with sieve-element cytoskeleton action). LatA pretreatment had no effect on the rate of forisome dispersion in response to distant burning in *Vicia faba* (n = 84, results not shown).

**Fig 7:**
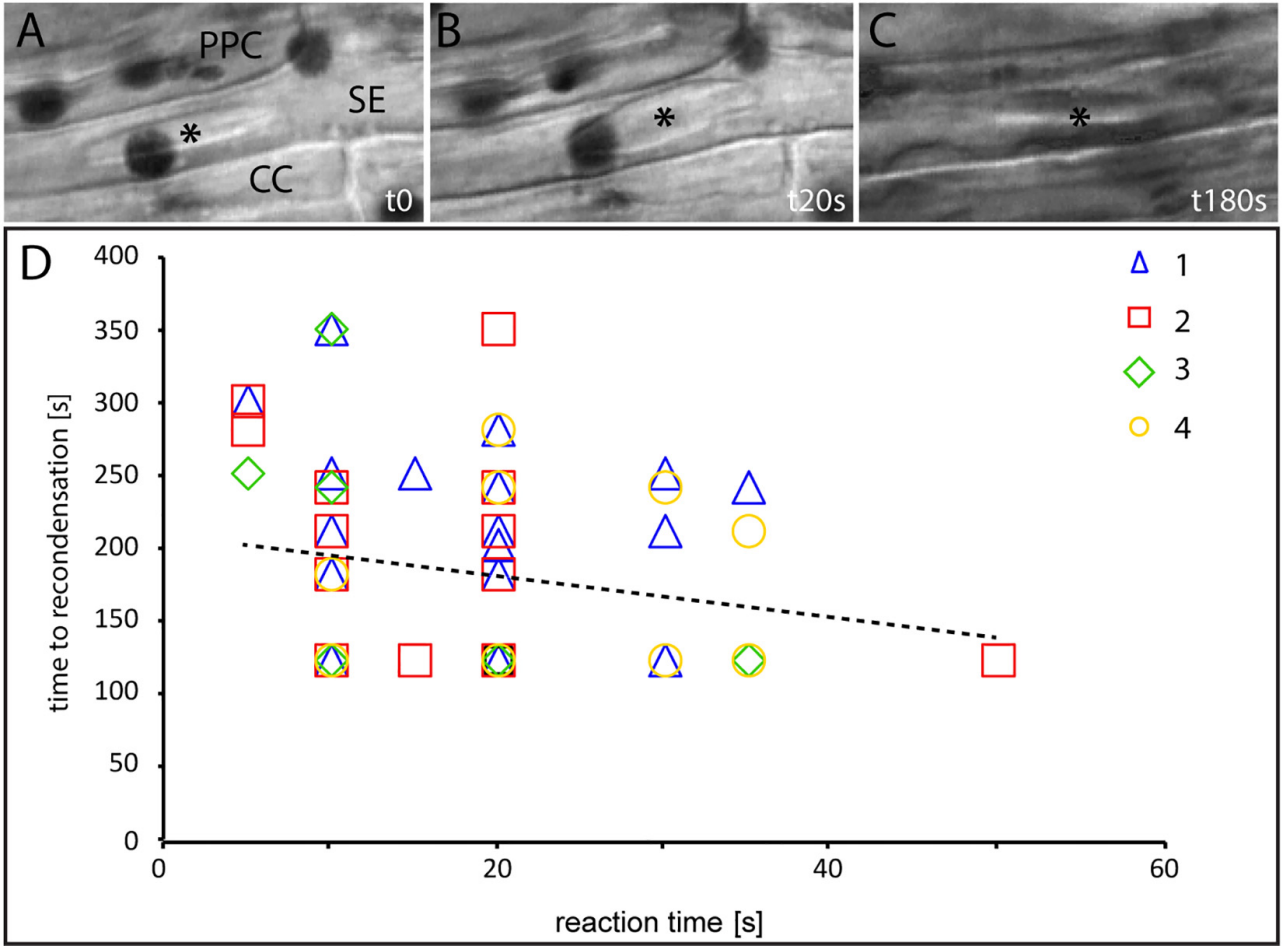
A-C Dispersion and re-condensation of a forisome in a LatA pretreated non-primed *Phaseolus vulgaris*plant in response to a remote heat stimulus. D. Relationship between dispersion and re-condensation times. The stippled line indicates the relationship between dispersion and re-condensation time.

By contrast, LatA pre-treatment considerably changed the reaction pattern of basal forisomes in response to burning in non-primed *Phaseolus vulgaris* plants (Tables 2 and 3). LatA pretreatment had a severe impact on the capacity of movement. None of the basal forisomes (n = 74) did move after burning (Table 3), but dispersed instead (Fig 7A–7C). The dispersion percentages reached even higher values than those obtained with primed control plants (cf. Fig 4F). As with other dispersion reactions, there was no correlation between reaction and re-condensation times of these forisomes (Fig 7D; correlation coefficient R< 0,005).

**Table 3:** Reactivity of basal forisomes pretreated with LatA in response to remote burning of intact non-primed *Phaseolus vulgaris* plants as related to its position, without prior priming. “Reaction” includes both dispersion and movement.

	number of replicates	dispersion [%]	movement [%]	no reaction [%]	reaction [%]
**1**	**35**	**86** (n = 30)	**0**	**14** (n = 5)	**86** (n = 30)
**2**	**19**	**74** (n = 14)	**0**	**26** (n = 5)	**74** (n = 14)
**3**	**11**	**55** (n = 6)	**0**	**45** (n = 5)	**55** (n = 6)
**4**	**15**	**67** (n = 10)	**0**	**33** (n = 5)	**67** (n = 10)

In control experiments, dimethyl sulfoxide (DMSO) at the concentrations used here to dissolve LatA had no effect on forisome conformation changes (*Vicia faba* n = 6; *Phaseolus vulgaris* n = 12, results not shown).

## Discussion

### Relationship between location or position and forisome dispersion responsiveness

The distribution (i.e. the locations) of forisomes is quantitatively similar, but significantly different in sieve elements of *Vicia faba* and *Phaseolus vulgaris* (Fig 3). The positions of tailless and tailed forisomes at the respective locations are almost identical (Fig 3). About 30% of the forisomes are in contact with the sieve-element side facing the companion cell (Fig 3). Since the common interface between sieve element and companion cell amounts about 30% of the total sieve-element inner surface in transport phloem [30, 31], there seems no preference for forisome contacts with the sieve-element companion cell interface.

Visual inaccuracies due to using the confocal technique in the transmission mode prevented us to precisely observe if the forisome was docked onto a membrane structure. The significantly differential reactivity of forisomes in various positions (Fig 4) however demonstrates that the position assessment was largely correct. We refrained from employing stained forisomes (a CMEDA/CMFDA mixture; [20, 21]) which might have provided sharper images, because they displayed an aberrant dispersion behaviour in preliminary experiments. Thus, attachment or anchoring to membranes could not be established with certainty by microscopy. Therefore, we avoided the term “attachment” and preferred the vaguer term “contact”.

Reactivity of forisomes to remote stimuli has been related to the amount of Ca^2+^ released into the sieve-element lumen via Ca^2+^-permeable channels during passage of an electropotential wave [23]. The reactivity (expressed as the dispersion rate) of the basally located forisomes exceeds that of the others (Fig 4E and 4F) which concurs with a clustering of Ca^2+^-permeable channels in the sieve-plate area [21, 23]. However, it does not explain why the forisomes located at the upstream end are much less reactive (Fig 4E and 4F), although forisomes at the sieve-element ends are both positioned near a sieve plate. It infers that the Ca^2+^-permeable channels are more densely aggregated at the downstream side of the sieve plate which finds confirmation in pictures, where Ca^2+^ channels inside the sieve tubes are stained by DM-BODIPY-DHP (lower inset Fig 4J in [21]). As exemplified by the basally located forisomes (Fig 4E and 4F), contacts with sieve-element membrane structures are decisive for the degree of forisome reactivity. This renders credence to the view that Ca^2+^ hotspots in the vicinity of forisome tips are required for dispersion [23].

Dispersion and re-condensation times do not appear correlated as observed in a less detailed study [21]. This is logical given the fact that dispersion and re-condensation depend on different, only weakly interdependent processes [24]. Dispersion is due to gating of Ca^2+^-permeable channels [21, 23], whereas re-condensation presumably relies on the activity of Ca^2+^-ATPases [32, 33].

### Arguments in favour of actin-mediated mobility of bushbean forisomes

About 20 to 30% of the forisomes are not located at the downstream end of sieve elements (Figs 2 and 3). This seems inconsistent with the view that forisomes are free-floating and, hence, would be pressed against the downstream sieve plate by mass flow. One may argue, however, that free-floating forisomes dragged along by mass flow get stuck behind obstacles in the mictoplasm [15] which might explain the forisome presence throughout the sieve element. This interpretation may hold for a few forisomes—the anchoring does not seem excessively tight—but does not explain the uneven forisome distribution over the sieve element [15] (Figs 2 and 3). The preference for the upstream location in comparison with the paucity of forisomes in the central region (Fig 2) speaks for some mode of attachment, unless preferential forisome residence near sieve plates results from opposite fluxes in sieve tubes. However, phloem mass flow in apical direction is unlikely in petioles of source leaves.

All in all, upstream localization seems to indicate that forisomes are “anchored” in some way, preferably in the vicinity of Ca^2+^ hotspots [24]. Some form of forisome attachment is corroborated by forisome displacement in *Phaseolus* in response to remote heating (Fig 5). Forisome movement vice versa points to a mechanism for active forisome movement which requires anchoring to structural sieve-element components. As forisomes themselves do not seem to have the instruments for mobility, movement may depend on an interaction between forisomes and sieve-element components via structural links. These connections are likely coupled to the sieve-element cytoskeleton as shown by the full inhibition of forisome displacement by LatA (Tables 1–3).

One could argue that the reversible mobility of bushbean forisomes is purely passive due to reverse fluid movements—pressure followed by retraction—induced by distant heating. Purely passive fluid-propelled movement of bushbean forisomes in response to heat stimuli, however, is incompatible with the immobility of broadbean forisomes in response to heat pulses and the elimination of the mobility of bushbean forisomes in the presence of LatA. In conclusion, the differential mobility of bushbean and broadbean forismes is inconsistent with passive massive-flow driven movement.

Admittedly, the presence of a cytoskeleton in SEs is a matter of debate. Electron microscopic studies in the 20^th^ century could not identify a cytoskeletal system in sieve elements [34]. It is hard to conceive, however, how the intense macromolecular exchange between SEs and CCs [35] can take place without a cytoskeleton crossing the Plasmodesm-pore units (PPUs) s like in other plasmodesmata [36]. A cytoskeleton extending from CCs into SEs would also provide a conveyer belt for sorting macromolecules inside the SE for local and distant destinations [37]. An actin network in SEs was visualized by fluorescent phalloidin and immunocytochemistry [29]. Conversely, Cayla *et al*. [38] could not find actin filaments in SEs of Arabidopsis, but they do not exclude that the fluorescent fABD2:GFP constructs expressed in CCs are too large to pass PPUs or that the density of actin filaments in SEs is low. A coarse-meshed network of thin actin filaments in SEs might indeed explain their apparent absence in EM pictures.

### Questions regarding presumptive forisome anchoring and mobility

Despite the first glimpses on their remarkable behaviour, the nature of dispersion and mobility of *Phaseolus* forisomes is fraught with questions that remain to be answered:

1 It is unclear as how forisomes find their original position after a stimulus. Tailless forisomes that are stimulated below a certain Ca^2+^ threshold do not disperse, but start wiggling for a while [23]. A small change of orientation happens to dispersing *Vicia* forisomes (Fig 5A–5E). Both movements of broadbean forisomes may be interpreted as a release of anchors which are not connected with a frame for movement. Subsequent to their reaction, *Vicia* forisomes are able to regain their original position (Fig 5A–5E). The same applies to *Phaseolus* forisomes, the return of which to the original position must be more complex than in *Vicia* given the extensive longitudinal movement (Fig 5F–5O). *Phaseolus* forisomes may be anchored to a motive apparatus, perhaps via the tails, which have a SEO composition distinct from the rest of the forisome body [8, 13]. Forisome tails do not disperse in response to Ca^2+^ supply (Fig 1) [19] and are only weakly fluorescent in forisomes in which certain SEO-proteins are GFP-tagged [8].
2 Once *Vicia* forisomes have reached the condensed state following the preparation procedure, they are ready to disperse in response to remote heat shocks. By contrast, *Phaseolus* forisomes require a precedent slight mechanical shock to become much more reactive in terms of dispersion. On the other hand, heat shocks induce forisome movement in non-primed *Phaseolus* plants. It appears that both dispersion and movement require input of Ca^2+^ ions. The crux for the differential behaviour of bushbean forisomes may lie in the availability of Ca^2+^ and competition for Ca^2+^ by respective binding sites. Application of LatA fully impairs longitudinal movement of tailed condensed forisomes, which disperse at the rates usual for non-moving forisomes when the capacity of movement is blocked. This behaviour may be interpreted in terms of competition. If Ca^2+^ cannot be invested into movement due to the LatA-induced inactivity of actin, it is available for alternative binding sites needed for dispersion. The interpretation also implies that the Ca^2+^ threshold for activation of forisome movement exceeds that needed for triggering of dispersion.
3 The manual touch as an inductor of “priming” may provoke electropotential waves akin to touch-triggered [28, 39, 40] and vibration-generated [41] action potentials, which are associated with Ca^2+^ influx into sieve tubes along the pathway. They likely prepare the plant to potential attacks of herbivores [41].

The “priming cue” may actually suppress Ca^2+^ influx after a heat shock. The slight touch may evoke an electropotential wave causing a suboptimal Ca^2+^ influx. A second electropotential wave triggered by the heat shock during the refractive period of the touch-induced action potential [42, 43] may cause less Ca^2+^ influx than in “non-primed” plants. As an effect of “priming”, the Ca^2+^ level may become sufficient for dispersion, but falls short to reach the threshold for forisome movement. A critical Ca^2+^ level for forisome movement also explains why merely tailed down-stream forisomes (39% of 95 forisomes) move in upstream direction, while a few forisomes located at the centre move acropetally (6% of 34 forisomes) and none of the apical forisomes did (0% of 48 forisomes). As argued above, there may be an base-to-apex Ca^2+^ gradient in sieve elements [21] as exemplified by forisome reactivity (Fig 4) after passage of an electropotential wave.

4 At first glance, it is puzzling, why the effect of LatA application on forisome dispersion differs between distant heat shocks (control level of dispersion with LatA, this paper) or local cold shocks (suppression of dispersion with LatA, [29]). On second thoughts, the reason may be obvious: in cold shocks gating of Ca^2+^ channels is strongly modulated by actin [29], whereas actin is less involved in the long-distance propagation of electropotential waves [24].
5 Temporary occlusion of the sieve tube turned out to be a major function of forisome dispersion in intact sieve elements [16, 22, 24]. In this frame, it is hard to conceive what the function of non-dispersed forisome movement could be.

### Is forisome mobility related to the plant species or to presence of tails?

Since the present experiments have been carried out with just two species for reasons of work-load reduction, the question arises as whether forisome mobility is a species-specific trait or is rather associated with the presence of tails. In contrast to the forisome body that contains SEO subgroup 1 proteins [44], forisome tails are composed of proteins of the SEO subgroup 2 [13, 44] and do not disperse in response to Ca^2+^ [8, 19]. They might react to Ca^2+^ supply in another manner. It has been speculated that forisomes detach from anchoring sites under the control of Ca^2+^ during dispersion [23, 24]. On top of that, Ca^2+^ might be involved in forisome movement along actin filaments. On the basis of provisional circumstantial evidence, we believe that tails are decisive for forisome movement.

## Supporting Information

S1 Fig A-I. Z-stack illustration of *Phaseolus vulgaris* phloem tissue using the CLSM transmission mode shows a series of 2μm sections. The sieve tube diameter is approximately 18μm. Direction of flow is from right to left. A. SE = sieve element; SP = sieve plate; CC = companion cell. A,B. The forisome is marked by an asterisk, C. The tails are marked by arrowheads. Scale bar = 10μm.
(TIF)

S2 Fig A-E. Z-stack illustration of *Vicia faba* phloem tissue using CLSM the transmission mode shows a series of 4μm sections. The sieve tube diameter is approximately 20μm. Direction of flow is from right to left. A. SE = sieve element; SP = sieve plate; CC = companion cell. B. The forisome is marked by an asterisk. Scale bar = 10μm.
(TIF)

S3 Fig Movie showing movement of a tailed forisome in *Phaseolus vulgaris* in the middle of the screen after a distant heat shock. (WMV)

S4 Fig Isolated tailless (A,B) and tailed (C,D) forisomes (without Ca^2+^ A and C; with Ca^2+^ supply B and D). **A,B *Vicia*** *faba*, C,D *Phaseolus vulgaris*. *Isolation of forisomes*: Forisomes were isolated from *Vicia faba* and *Phaseolus vulgaris* phloem tissue according to Knoblauch et al. 2003. Isolation media containing 10 mM Tris (pH 7.3), 50 mM potassium chloride and ethylene-diaminetetraacetic acid (EDTA) concentrations of 2 or 10 mM were previously outgassed and covered with argon gas. 1 mM sodium sulfite was added to the isolation medium to suppress oxygen effects on forisomes. The cortex of the stems of 4- to 7-week-old *Vicia faba* or *Phaseolus vulgaris* plants was carefully pulled off, and phloem was scraped off with a scalpel. The phloem shreds were transferred to 2 ml of forisome isolation medium containing 10 mM EDTA. After 30 min of incubation, the phloem material was homogenized in liquid nitrogen and transferred to 4 ml of 2 mM EDTA solution. After filtration of solubilized plant material through a 60 μm mesh filter freshly isolated forisomes were used for *in vitro* studies. To observe forisome reactions, a drop of 10 μl 2mM EDTA solution containing forisomes was transferred to a microscope slide. After having a spindle shape forisome in focus this drop was exchanged successively against 10 mM calcium chloride solution to induce forisome dispersion.
(TIF)
