# Supplementary figures and images for: Similar Intracellular Location and Stimulus Reactivity, but Differential Mobility of Tailless (Vicia faba) and Tailed Forisomes (Phaseolus vulgaris) in Intact Sieve Tubes
Source: PLoS One. 2015 Dec 1;10(12):e0143920. doi: 10.1371/journal.pone.0143920 (PMC4666637; doi:10.1371/journal.pone.0143920)

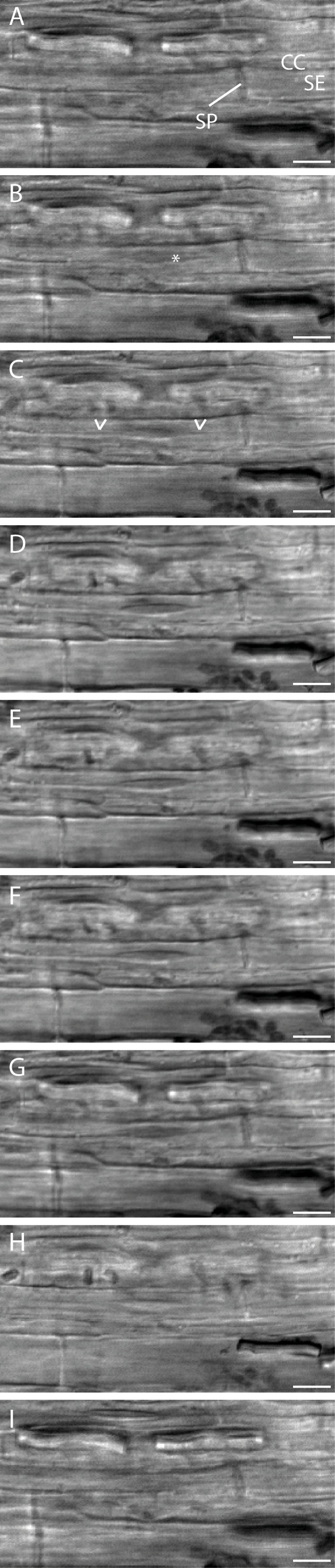

Supplement: S1 Fig — The sieve tube diameter is approximately 18μm. Direction of flow is from right to left. A. SE = sieve element; SP = sieve plate; CC = companion cell. A,B. The forisome is marked by an asterisk, C. The tails are marked by arrowheads. Scale bar = 10μm. (TIF) [file pone.0143920.s001.tif]

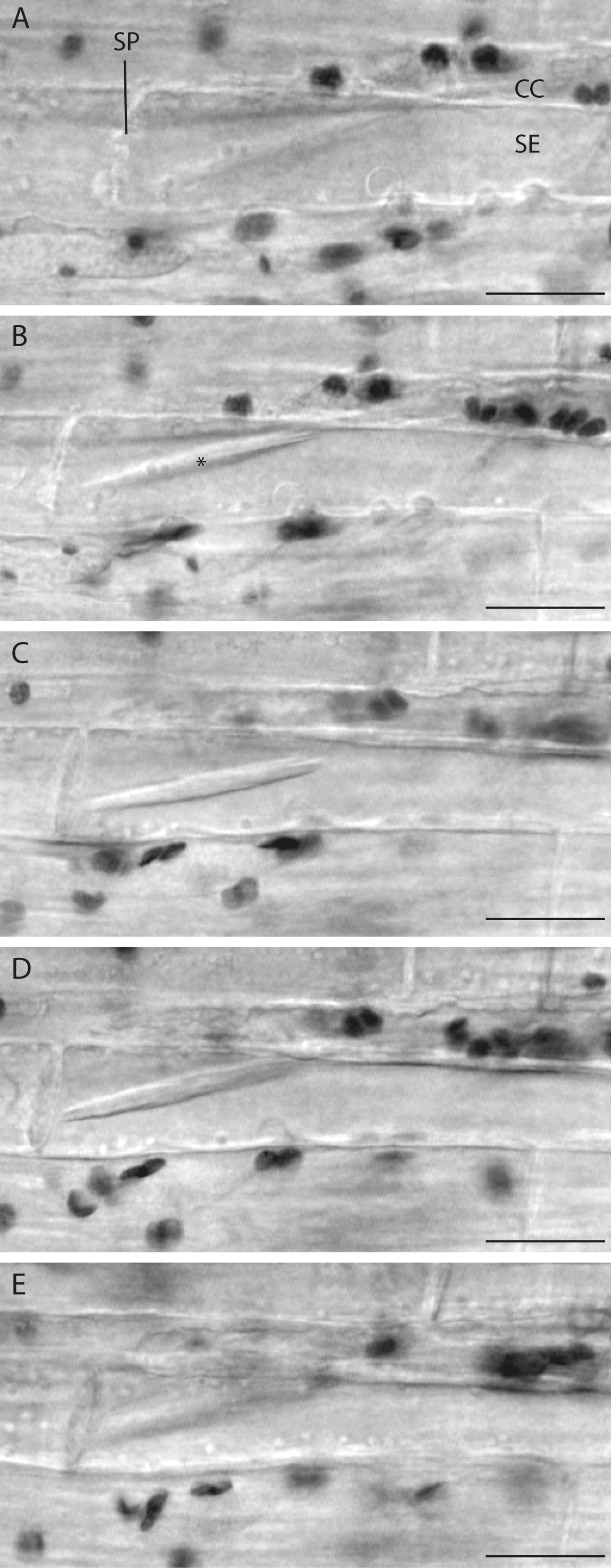

Supplement: S2 Fig — The sieve tube diameter is approximately 20μm. Direction of flow is from right to left. A. SE = sieve element; SP = sieve plate; CC = companion cell. B. The forisome is marked by an asterisk. Scale bar = 10μm. (TIF) [file pone.0143920.s002.tif]

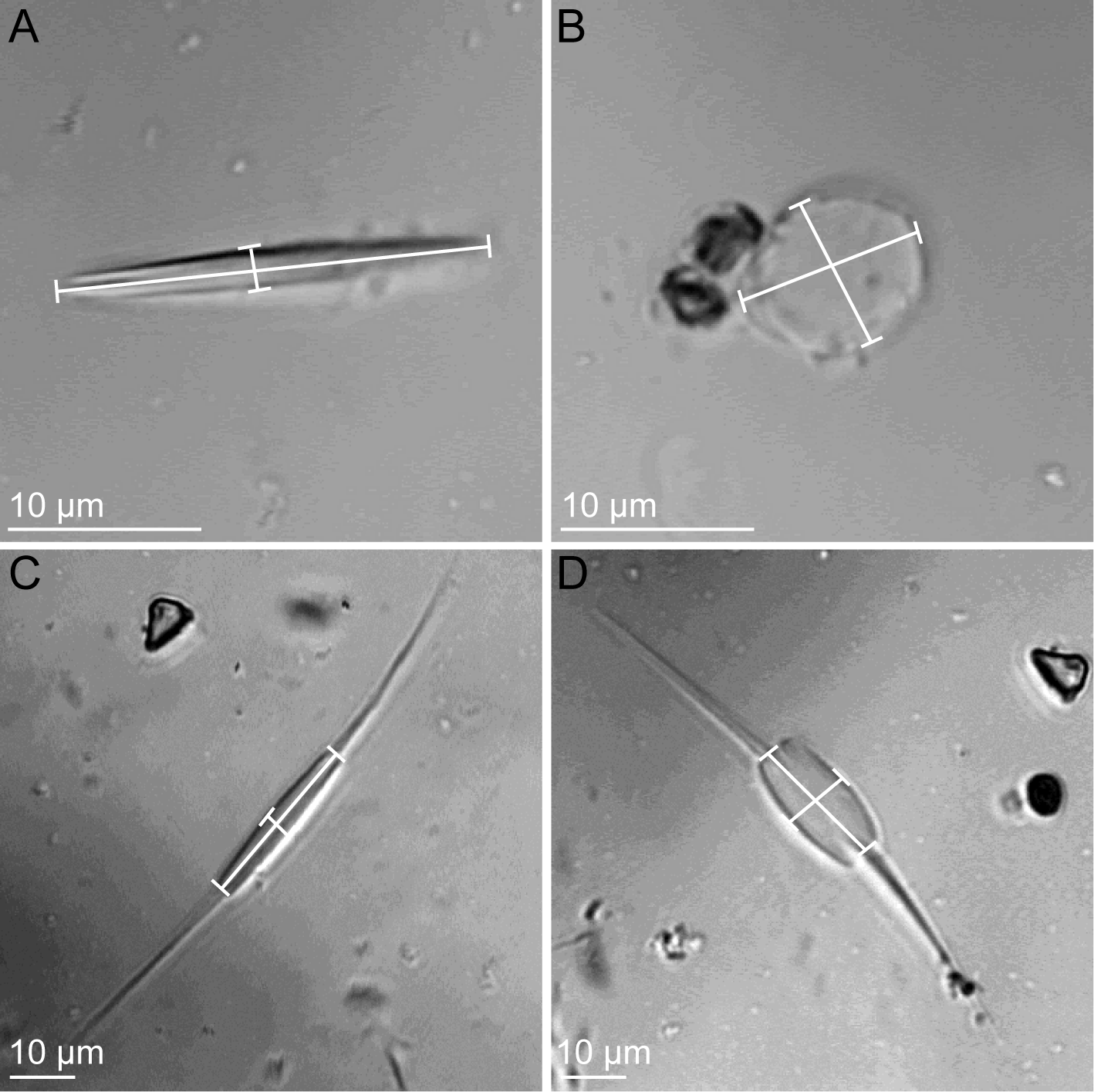

Supplement: S4 Fig — A,B Vicia faba, C,D Phaseolus vulgaris. Isolation of forisomes: Forisomes were isolated from Vicia faba and Phaseolus vulgaris phloem tissue according to Knoblauch et al. 2003. Isolation media containing 10 mM Tris (pH 7.3), 50 mM potassium chloride and ethylene-diaminetetraacetic acid (EDTA) concentrations of 2 or 10 mM were previously outgassed and covered with argon gas. 1 mM sodium sulfite was added to the isolation medium to suppress oxygen effects on forisomes. The cortex of the stems of 4- to 7-week-old Vicia faba or Phaseolus vulgaris plants was carefully pulled off, and phloem was scraped off with a scalpel. The phloem shreds were transferred to 2 ml of forisome isolation medium containing 10 mM EDTA. After 30 min of incubation, the phloem material was homogenized in liquid nitrogen and transferred to 4 ml of 2 mM EDTA solution. After filtration of solubilized plant material through a 60 μm mesh filter freshly isolated forisomes were used for in vitro studies. To observe forisome reactions, a drop of 10 μl 2mM EDTA solution containing forisomes was transferred to a microscope slide. After having a spindle shape forisome in focus this drop was exchanged successively against 10 mM calcium chloride solution to induce forisome dispersion. (TIF) [file pone.0143920.s004.tif]
